# Protein phosphatase AP2C1 negatively regulates basal resistance and defense responses to *Pseudomonas syringae*

**DOI:** 10.1093/jxb/erw485

**Published:** 2017-01-06

**Authors:** Volodymyr Shubchynskyy, Justyna Boniecka, Alois Schweighofer, Justinas Simulis, Kotryna Kvederaviciute, Michael Stumpe, Felix Mauch, Salma Balazadeh, Bernd Mueller-Roeber, Freddy Boutrot, Cyril Zipfel, Irute Meskiene

**Affiliations:** 1 Max F. Perutz Laboratories, University and Medical University of Vienna, Dr Bohrgasse 9, A-1030 Vienna, Austria; 2 Institute of Biotechnology (IBT), University of Vilnius, Saulėtekio al. 7, LT-10257 Vilnius, Lithuania; 3 Department of Biology, Chemin du Musée 10, CH-1700 Fribourg, Switzerland; 4 Max-Planck-Institute for Molecular Plant Physiology, Golm and University of Potsdam, D-14476, Germany; 5 The Sainsbury Laboratory, Norwich Research Park, Norwich NR4 7UH, UK; 6 Department of Ecogenomics and Systems Biology, University of Vienna, Althanstrasse 14, A-1090 Vienna, Austria

**Keywords:** Callose, defense genes, MAPK, MAPK phosphatase, PAMP, PP2C phosphatase, *Pseudomonas syringae*, salicylic acid, transcription factors.

## Abstract

Mitogen-activated protein kinases (MAPKs) mediate plant immune responses to pathogenic bacteria. However, less is known about the cell autonomous negative regulatory mechanism controlling basal plant immunity. We report the biological role of *Arabidopsis thaliana* MAPK phosphatase AP2C1 as a negative regulator of plant basal resistance and defense responses to *Pseudomonas syringae*. AP2C2, a closely related MAPK phosphatase, also negatively controls plant resistance. Loss of AP2C1 leads to enhanced pathogen-induced MAPK activities, increased callose deposition in response to pathogen-associated molecular patterns or to *P. syringae* pv. *tomato* (*Pto*) DC3000, and enhanced resistance to bacterial infection with *Pto.* We also reveal the impact of AP2C1 on the global transcriptional reprogramming of transcription factors during *Pto* infection. Importantly, *ap2c1* plants show salicylic acid-independent transcriptional reprogramming of several defense genes and enhanced ethylene production in response to *Pto*. This study pinpoints the specificity of MAPK regulation by the different MAPK phosphatases AP2C1 and MKP1, which control the same MAPK substrates, nevertheless leading to different downstream events. We suggest that precise and specific control of defined MAPKs by MAPK phosphatases during plant challenge with pathogenic bacteria can strongly influence plant resistance.

## Highlight

During *Pseudomonas syringae* infection the MAPK phosphatase AP2C1 negatively regulates plant resistance and modulates defense responses, such as changing hormone levels, callose deposition and transcriptional reprogramming.

## Introduction

The ability to grow in an environment full of potentially pathogenic microbes is very important for plant survival. Plants recognize pathogen-associated molecular patterns (PAMPs), such as flagellin or elongation factor Tu (EF-Tu), via plasma membrane-localized pattern recognition receptors (PRRs) (Macho and Zipfel, 2014). This recognition rapidly activates a signaling network of mitogen-activated protein kinases (MAPKs) (Meng and Zhang, 2013), which induces a basal level of immunity called PRR-triggered immunity (PTI, also known as pattern- or PAMP-triggered immunity) (Tena *et al.*, 2011; Rasmussen *et al.*, 2012; Meng and Zhang, 2013; Couto and Zipfel, 2016). MAPKs play an important role in PTI, but how they are negatively regulated by phosphatases is less understood.

Arabidopsis MAPKs MPK3, 4 and 6 are central for the regulation of several basal defense responses, including pathogen-induced gene expression and the production of plant stress hormones, as well as antimicrobial compounds (Tena *et al.*, 2011; Rasmussen *et al.*, 2012; Meng and Zhang, 2013). Phosphorylation of the MAPK motif ‘pTEpY’ by MAPK kinases (MAPKKs) is essential for their activation, whereas MAPK inactivation relies on different types of protein phosphatases to dephosphorylate this motif. Protein tyrosine (PTP) and dual specificity (DSP) phosphatases dephosphorylate phospho-tyrosines (pY) or both phospho-threonines (pT) and pY, respectively (Bartels *et al.*, 2010; Caunt and Keyse, 2013). Ser/Thr protein phosphatases of type 2C (PP2C) target pT in the ‘pTEpY’ loop (Meskiene *et al.*, 2003; Fuchs *et al.*, 2013). These types of phosphatases have been found to control MAPK signaling during plant defense. PTP1 and the DSP MKP1 have genetically overlapping roles in suppressing plant defense responses via inactivation of MPK3 and MPK6 (Bartels *et al.*, 2009). MKP1 negatively regulates a subset of PAMP-regulated genes, and MPK6-dependent resistance to the virulent bacterial pathogen *Pseudomonas syringae* pv. *tomato* (*Pto*) DC3000 (Bartels *et al.*, 2009; Anderson *et al.*, 2011, 2014). MKP2 interacts with MPK6 and controls the hypersensitive response in plants (Lumbreras *et al.*, 2010). Structurally and functionally different from PTP/DSP, phosphatases of the PP2C type such as Arabidopsis AP2C1, 2, 3 and 4 (Schweighofer *et al.*, 2004) also dephosphorylate MAPKs (Umbrasaite *et al.*, 2010). The alfalfa AP2C1 ortholog MP2C inactivates the MPK6 alfalfa ortholog SIMK by dephosphorylating pT in the ‘pTEpY’ motif (Meskiene *et al.*, 2003).

Among the 80 Arabidopsis PP2C family members, AP2C proteins feature a kinase interaction motif (KIM) (Fuchs *et al.*, 2013), which mediates interaction with MAPKs and is highly similar to the KIM motif found in mammalian MAPK phosphatases (Farooq and Zhou, 2004). The specificities for interactions between AP2Cs and MAPKs have been demonstrated, and extensive yeast two-hybrid screenings with AP2Cs repeatedly isolated MPK6 (Schweighofer *et al.*, 2007; Umbrasaite *et al.*, 2010). Interaction between AP2Cs and MPK3, 4 and 6 has been confirmed by bimolecular fluorescence complementation (BiFC) (Schweighofer *et al.*, 2007; Brock *et al.*, 2010; Umbrasaite *et al.*, 2010) and co-immunoprecipitation (Schweighofer *et al.*, 2007). AP2C1 modulates wound- and PAMP-induced MAPK activities (Schweighofer *et al.*, 2007; Galletti *et al.*, 2011), and its overexpression impairs wound-induced ethylene (ET) production (Schweighofer *et al.*, 2007) and plant immunity to the necrotrophic fungus *Botrytis cinerea* (Schweighofer *et al.*, 2007; Galletti *et al.*, 2011). PAMP perception highly upregulates the expression of *AP2C1* and its closest homolog *AP2C2* in Arabidopsis plants (Navarro *et al.*, 2004; Zipfel *et al.*, 2006). However, their role in plant responses to hemibiotrophic pathogens such as *Pseudomonas syringae* remains unclear. We approach this question in the current study.

Many of the PAMP-induced transcriptional alterations (Zipfel *et al.*, 2004, 2006) are downstream of MAPKs projected for resetting the cellular processes for defense responses (Meng and Zhang, 2013). PAMPs and pathogens transiently activate MPK3, 4, 6, and 11 (Nühse *et al.*, 2000; Asai *et al.*, 2002; Bethke *et al.*, 2012), which leads to phosphorylation of proteins, including transcription factors (TFs) (Liu and Zhang, 2004; Bethke *et al.*, 2009; Popescu *et al.*, 2009; Andreasson and Ellis, 2010; Mao *et al.*, 2011; Lassowskat *et al.*, 2014). The ability of MAPKs to phosphorylate different TFs, which are also partially shared between MAPKs (Popescu *et al.*, 2009), adds to the complexity in dissecting defined roles of MAPKs. Direct targets of MAPKs are WRKY TFs, which regulate the expression of many pathogen-responsive genes. MPK3 and MPK6 phosphorylate WRKY33, which stimulates the expression of *PHYTOALEXIN DEFICIENT 3* (*PAD3*) (Mao *et al.*, 2011). *PAD3* encodes an enzyme required for the synthesis of the antimicrobial compound camalexin (Qiu *et al.*, 2008). WRKY33 is also involved in MPK3- and MPK6-induced expression of *ACS2* and *ACS6*, which encode enzymes involved in ET biosynthesis (Li *et al.*, 2012). Direct phosphorylation of ACS2 and ACS6 by MPK3 and MPK6 and ensuing stabilization of these proteins enhances ET biosynthesis (Liu and Zhang, 2004; Joo *et al.*, 2008; Han *et al.*, 2010). ET is important for PTI amplification and maintenance by replenishment of ligand-free FLS2 and also by activation of signaling mediated by PEPR1/2 to induce immunity to pathogens (Boutrot *et al.*, 2010; Liu *et al.*, 2013; Tintor *et al.*, 2013; Zipfel, 2013). ET promotes the release of ERF104 from MPK6, presumably to access target genes for plant defense (Bethke *et al.*, 2009). PAMP perception by PRRs leads to production of salicylic acid (SA) (Mishina and Zeier, 2007), which plays a central role in PTI (Dempsey *et al.*, 2011). MPK3 and MPK6 positively regulate SA signaling (Zhang *et al.*, 2007*b*), and MPK4 has been identified as a positive regulator of PTI (Zhang *et al.*, 2012). An SA marker gene, *PR1* (Ward *et al.*, 1991), is also regulated by alternative SA-independent mechanisms, such as the one induced by sustained MPK3 activation (Tsuda *et al.*, 2013).

Transcriptional regulation plays an important role in plants (Mitsuda and Ohme-Takagi, 2009). For global TF analysis, qRT-PCR is a sensitive and preferred method (Czechowski *et al.*, 2004; Rauf *et al.*, 2013), but TF profiling by qRT-PCR has not yet been applied to plant–pathogen studies in respect to cell signaling. In our study, we employed an Arabidopsis whole genome qRT-PCR platform for 1880 TFs and 137 defense response genes (Brotman *et al.*, 2012) aiming to identify genes involved in *AP2C1* regulated cell signaling ensuing infection by pathogen *Pto* DC3000.

The present study demonstrates a strongly enhanced resistance to *Pto* DC3000 in the *ap2c1* mutant, providing an important genetic model for investigating the basis for the induction of enhanced plant resistance. We address the regulatory mechanism of MAPK activities by AP2C1 and relate activation of MAPKs with distinct cellular responses during pathogen/PAMP-induced signaling, expression of TFs and defense genes, as well as ethylene and callose accumulation. These findings highlight the significance of the MAPK pathway regulation by AP2C1 phosphatase for plant defense.

## Materials and methods

### Plant material

For this study the following mutant lines of *Arabidopsis thaliana* (L.) Heynh. (Col-0 accession) were used: *ap2c1* (SALK_065126) (Schweighofer *et al.*, 2007), *ap2c2* (GABI-Kat_316F11) (Umbrasaite *et al.*, 2010), *mkp1* (Bartels *et al.*, 2009), *mpk3-1* (SALK_151594), *mpk6-2* (SALK_073907), *fls2c* (SAIL_691_C4) (Zipfel *et al.*, 2004), *efr-1* (SALK_044334) (Zipfel *et al.*, 2006), *fls2c efr-1 cerk1-2* (*fec*) (Gimenez-Ibanez *et al.*, 2009*b*), 35S-AP2C1-GFP, and *ap2c1* complemented line (644) (Schweighofer *et al.*, 2007). *ap2c1 mpk3* and *ap2c1 mpk6* double mutants were created by crossing single mutants and verified by PCR in the F2 generation [*ap2c1* T-DNA (forward: 5′-TGGTTCACGTAGTGGGCCATCG-3′ and reverse: 5′-CATCAGACGAGCCTCGTGAAGCAGATAAATCG-3′); AP2C1 (forward: 5′-TCGGCCGCTGTGGCTGCG -3′ and reverse: 5′-CATCAGACGAGCCTCGTGAAGCAGATAAATCG-3′; *mpk3* T-DNA (forward: 5′-GCTTGGCACACCGACAGAATCT-3′ and reverse: 5′-TGGTTCACGTAGTGGGCCATCG-3′); MPK3 (forward: 5′-GCTTGGCACACCGACAGAATCT-3′ and reverse: 5′-ACCGTATGTTGGATTGAGTGCTATG-3′); *mpk6* T-DNA (forward: 5′-GGCATCGTTTGTTCGGCTATG-3′ and reverse: 5′- TGGTTCACGTAGTGGGCCATCG -3′); MPK6 (forward: 5′-GGCATCGTTTGTTCGGCTATG-3′ and reverse: 5′- GATCT CGTCCAGGGAAGAGTG-3′)]. Plants on soil were grown at 21–22 °C with an 8 h light/16 h dark photoperiod in environmentally controlled growth chambers. For growth under sterile conditions seeds were surface sterilized (1 min in 96% ethanol, 5 min in 7.5% NaOCl–0.01% Triton X-100, five washes with sterile water). Seedlings were grown on plates containing half-strength Murashige and Skoog (MS) medium (Duchefa), 1% sucrose, and 0.7% Bacto agar (Invitrogen) at 22 °C with a 10 h light/14 h dark photoperiod. For ethylene measurements seeds were spread in glass vials containing 15 ml half-strength MS medium (Duchefa), 1% sucrose, and 0.7% Bacto agar (Invitrogen), and the glass vials were transferred to a growth chamber and kept in long day photoperiod (16 h light/8 h dark), at 50% humidity and 23 °C temperature.

### *Pseudomonas* infection and growth assay

Bacterial strains used in this study were *Pseudomonas syringae* pv. *tomato* DC3000 (*Pto* DC3000), *Pseudomonas syringae* pv. *tomato* DC3000 *ΔavrPto*/*ΔavrPtoB* (*Pto* DC3000 *ΔavrPto*/*ΔavrPtoB*), *Pseudomonas syringae* pv. *tomato* DC3000 *COR*^*−*^ (*Pto* DC3000 *COR*^*−*^). For bacterial enumeration assays, plants were sprayed with the strains (inoculum: 10^7^ cfu ml^−1^, OD_600_=0.02), in the presence of 0.001% (v/v) Silwet L-77, as described (Zipfel *et al.*, 2004). Sprayed plants were covered with a transparent plastic lid for the remaining time of the experiment. Bacterial titer was estimated 3 or 4 d after infection.

### PAMP treatment, protein extraction, SDS-PAGE and western blot

PAMP treatments were performed by spraying flg22 and elf18 elicitor peptides (Peptron, South Korea), which were dissolved in water to a concentration of 0.1 or 1 µM.

Total protein was extracted from frozen leaf tissue, subjected to SDS-PAGE, transferred to a polyvinylidene fluoride membrane (Millipore), and used for immunodetection as described (Meskiene *et al.*, 2003). Equal loading was checked by Ponceau S staining. Anti-phospho-p44/42 MPK (Thr202/Tyr204) antibody (Cell Signaling Technology), was used to detect doubly phosphorylated MAPKs. Antigen–antibody complexes were detected with horseradish peroxidase-conjugated anti-rabbit secondary antibody (Cell Signaling Technology) followed by chemiluminescence detection with SuperSignal West Pico chemiluminescent substrate (Pierce).

### Quantitative RT-PCR

Plants were grown for 4 weeks on soil and treated for 0, 4, and 48 h with *Pto* DC3000 (OD_600_=0.02). Total RNA was isolated with the RNeasy Plant Mini kit (Qiagen). DNase Turbo DNA-free (Ambion) was used for genomic DNA removal. Absence of genomic DNA was confirmed by qRT-PCR using intron-specific primers for the gene At5g65080 (forward: 5′-TTTTTTGCCCCCTTCGAATC-3′ and reverse: 5′-ATCTTCCGCCACCACATTGTAC-3′). RNA integrity was checked on 1% (w/v) agarose gel and concentration measured with a Nanodrop ND-1000 spectrophotometer (Thermo Scientific) before and after DNAse I digestion. cDNA was synthesized from 2 µg of total RNA using RevertAid-First Strand cDNA Synthesis Kit (Fermentas) with oligo-dT primers, according to the manufacturer’s instructions. The efficiency of cDNA synthesis was determined by qRT-PCR amplifications of control transcripts of the genes *ACTIN2* (At3g18780; forward: 5′-TTCCTCAGCACATTCCAGCAGAT-3′ and reverse: 5′-AACGATTCCTGGACCTGCCTCATC-3′), *GAPDH* 5′ region (At1g13440; forward: 5′-TCTCGATCTCAATTTCGCAAAA-3′ and reverse: 5′-CGAAACCGTTGATTCCGATTC-3′) and *GAPDH* 3′ region (At1g13440; forward: 5′-TTGGTGACAACAGGTCA AGCA-3′ and reverse: 5′-AAACTTGTCGCTCAATGCAATC-3′). qPCRs were performed either with SyberGreen master mix (Sigma-Aldrich) or with Power SYBER Green PCR Master Mix (Applied Biosystems). Gene expression was normalized to *ACTIN2*.

TF and defense-related expression profiling platforms were used as described previously (Czechowski *et al.*, 2004; Brotman *et al.*, 2012).

### ET measurements

Thirteen-day-old seedlings were treated with 1 µM elf18, 1 µM flg22, *Pto* DC3000 (OD_600_=0.04) or 10 mM MgCl_2_ solution. Two hundred microliters of solution was carefully placed on plant leaves as droplets. Glass vials with treated seedlings were capped air-tight and transferred to a plant growth chamber. Next day accumulated ethylene was measured using a Thermo Scientific FOCUS GC gas chromatograph with flame ionization detector and Supelco column (length 1.8 m, external diameter 3.2 mm, internal diameter 2.1 mm): 500 µl of gas sample was taken using a gas-tight micro syringe and loaded into the machine. Chromatograms were analysed using the Chrom-card program.

### SA and camalexin measurements

For the analysis of free and conjugated SA and camalexin, 0.25 g of leaves (with 200 ng of *o*-anisic acid) was extracted once with 2 ml of 70% methanol and once with 2 ml of 90% methanol by using a homogenizer (Polytron; Kinematica, Littau, Switzerland). After evaporation of the methanol from the combined extracts, trichloroacetic acid precipitation was performed. Free phenols and camalexin were extracted into cyclohexane–ethyl acetate (1:1). The remaining aqueous phase was submitted to acid hydrolysis in the presence of 4 M HCl at 80 °C for 1 h, and the liberated phenols were extracted into cyclohexane–ethyl acetate, as described above. For HPLC, the organic phase was evaporated, and the samples were resuspended in 85% phosphate buffer–15% acetonitrile. Chromatography was performed on a reverse phase HPLC column (ABZ+, 25 cm×4.6 mm; Supelco, Buchs, Switzerland) as described (Meuwly and Métraux, 1993).

### Callose deposition

Callose deposition was observed after spraying with a solution of 1 µM elf18 or *Pto* DC3000 (OD_600_=0.02) for 24 h as previously described (Luna *et al.*, 2011). Callose deposits were quantified using ImageJ software.

### Analysis of promoter motifs

Promoter motifs were identified using the Athena web-based research tool (http://www.bioinformatics2.wsu.edu/Athena). TF-binding frequency and enrichment for sub-selected promoters and TFs were calculated.

## Results

### Disease resistance to *P. syringae* is strongly enhanced in the Arabidopsis protein phosphatase mutant *ap2c1*

**Fig. 1.**
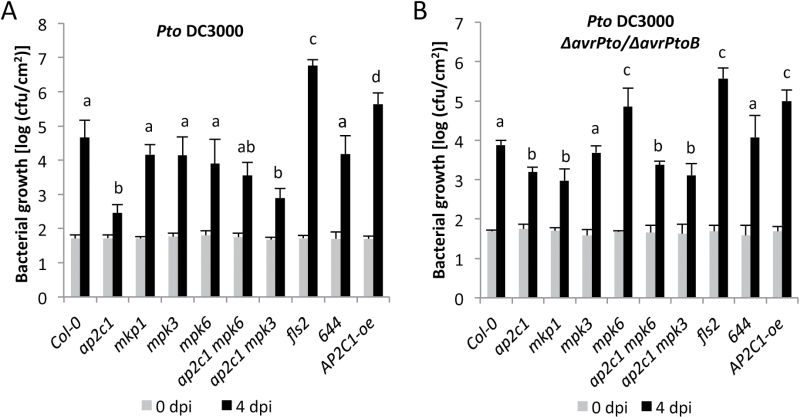
Susceptibility of plants to the pathogen *P. syringae.* Four-week-old plants were spray-infected with *P. syringae* pv. *tomato* (*Pto*) DC3000 (A) or *Pto* DC3000 *ΔavrPto/ΔavrPtoB* (B) and bacterial count measured at 4 days post-infection (dpi). Values shown are means±standard deviation (*n*=8) of one representative experiment from three independent repetitions. One-way ANOVA/Holm–Sidak: *a*≠*b*, *P*<0.01; *a*≠*c*, *P*<0.01; *a*≠*d*, *P*<0.01.

Previously, we demonstrated that the T-DNA knock-out mutant of the MAPK phosphatase *ap2c1* produces higher amounts of wound-induced jasmonic acid (JA) and is more resistant to phytophagous mites (*Tetranychus urticae*) (Schweighofer *et al.*, 2007). Plants with increased AP2C1 levels are compromised in basal and PAMP-induced resistance against the necrotrophic fungal pathogen *Botrytis cinerea* (Schweighofer *et al.*, 2007; Galletti *et al.*, 2011). To test whether AP2C1 regulates plant defense against bacterial hemibiotrophs, we infected *ap2c1* plants by spray-inoculation with the virulent bacterium *Pseudomonas syringae* pv. *tomato* DC3000 (*Pto* DC3000). Remarkably, we observed a strongly increased resistance in *ap2c1* compared with the wild-type Col-0 at 4 days post-inoculation (dpi) (Fig. 1A). This phenotype was observed neither in the null mutants *mpk3* or *mpk6*, nor in *mkp1*, whereas *fls2* plants were significantly more susceptible to *Pto* DC3000 (Fig. 1A and Supplementary Fig. S1 at *JXB* online), as reported previously (Zipfel *et al.*, 2004). The enhanced resistance to bacteria observed in *ap2c1* was reversed in transgenic lines complemented with *AP2C1-GFP* expressed under the native promoter (line 644) (Schweighofer *et al.*, 2007) (see Supplementary Fig. S1), indicating that the phenotype observed in *ap2c1* is caused by the lack of AP2C1. Moreover, plants overexpressing *AP2C1* (AP2C1-oe) were more susceptible to *Pto* DC3000 (Fig. 1). *Ap2c1* mutants were also more resistant to strains with attenuated virulence such as *Pto* DC3000 *ΔavrPto*/*ΔavrPtoB* (Fig. 1B), which lack the type III-secreted effectors AvrPto and AvrPtoB (Lin and Martin, 2005).

To assess the specificity of the enhanced plant resistance detected in *ap2c1*, we tested whether a similar phenotype could be observed in a T-DNA knock-out mutant line of the related MAPK phosphatase AP2C2 (Umbrasaite *et al.*, 2010), the closest paralog to AP2C1. Although we did not find significant changes in response to *Pto* DC3000 in *ap2c2* plants at 3 and 4 dpi (see Supplementary Fig. S1A), these plants, as well as *ap2c1* mutants, were more resistant to the weakly virulent *Pto* DC3000 *COR*^*–*^ (Supplementary Fig. S1B), which lack the jasmonic acid mimic coronatine (COR) (Brooks *et al.*, 2004).

Taken together, our data show that AP2C1 and to some extent AP2C2 are negative regulators of plant resistance to pathogenic bacteria.

### Activation of MAPKs by PAMPs and *Pto* DC3000 is enhanced in the *ap2c1* mutant

**Fig. 2.**
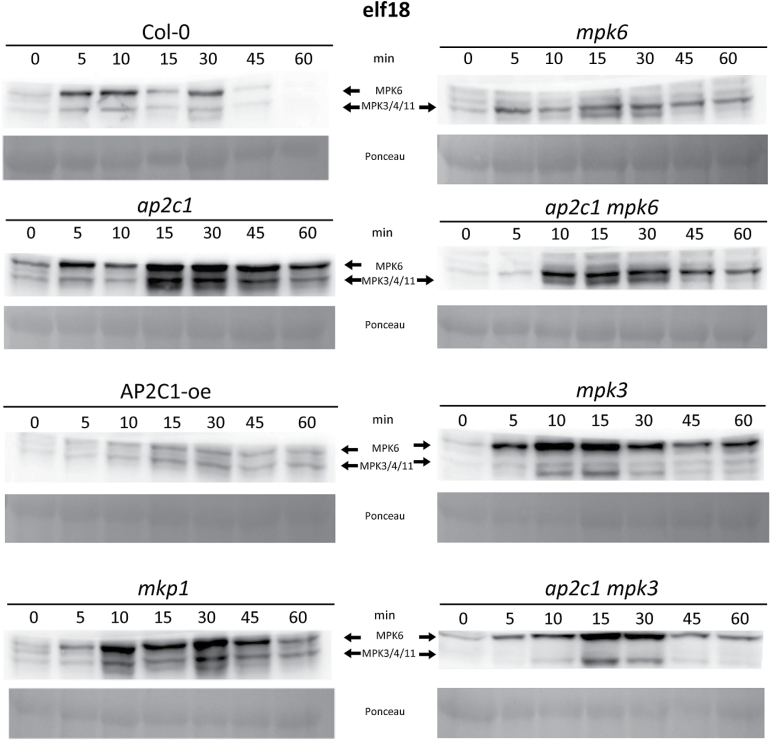
AP2C1 controls elf18-induced MAPK activation. Western blotting with p44/42 antibodies after application of 1 µM elf18 on seedlings. Profiling of MAPK activation by an immunological assay that detects phosphorylation of the MAPKs. MPK6 and MPK3/4/11 corresponding immunoreactive bands are indicated by arrows in the top panels. Ponceau staining was used to estimate equal loading (bottom panels).

To better understand the underlying mechanism of the enhanced bacterial resistance observed in *ap2c1* plants, we analysed the PAMP-induced activation of MPK3, MPK4 and MPK6, which are the substrates of AP2C1 (Schweighofer *et al.*, 2007; Galletti *et al.*, 2011). Our data show more pronounced and prolonged elf18- and flg22-induced MAPK activation in *ap2c1* than in Col-0 plants (Fig. 2 and Supplementary Fig. S2). Conversely, ectopic expression of *AP2C1* strongly inhibited elf18-induced MAPK phosphorylation (Fig. 2). Remarkably, the double mutant *ap2c1 mpk6* showed an MPK3/MPK4 phosphorylation that is much more pronounced than Col-0 and *mpk6* lines (Fig. 2). Similarly, the *ap2c1 mpk3* displayed a higher PAMP-induced MPK6 activity compared with the single mutants *ap2c1* or *mpk3*. The activation of MAPKs in *mkp1* plants was also enhanced and prolonged compared with Col-0 plants, but showed different kinetics and activity maximum.

**Fig. 3.**
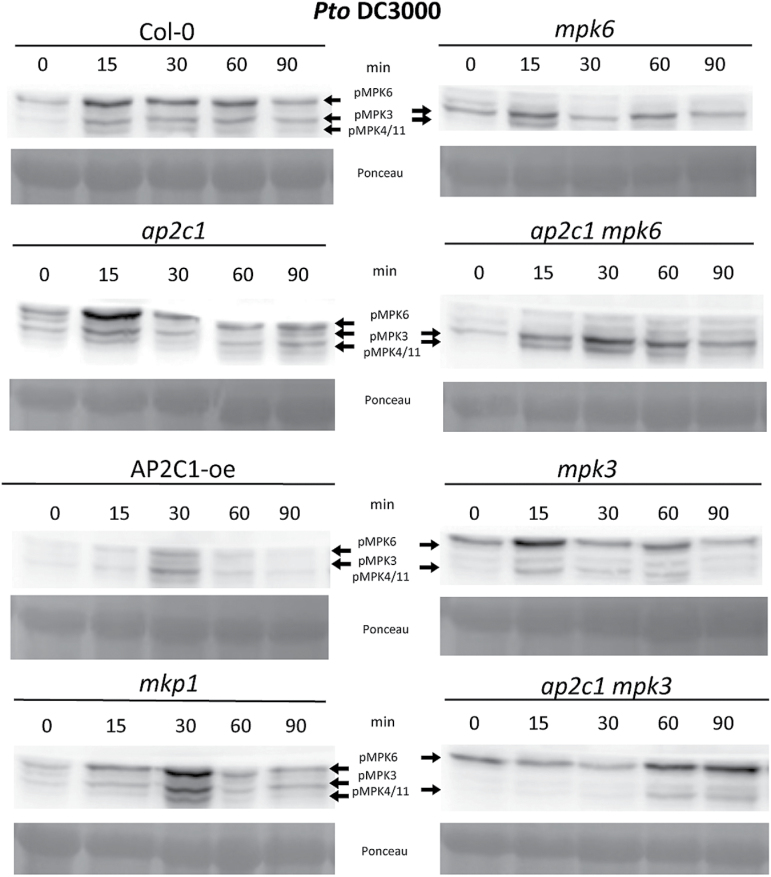
Analysis of MAPK activation in response to infection with *Pto* DC3000. Western blotting with anti-p44/42 antibodies. Bacteria induced activation of MAPKs in plants after treatment with *Pto* DC3000; OD_600_=0.02. The immunoreactive protein bands corresponding to respective MAPKs are indicated in the top panels. Ponceau staining was used to estimate equal loading (bottom panels).

To assess the effect of bacterial infection on MAPK activation, we characterized MAPK phosphorylation in response to *Pto* DC3000. In the *ap2c1* line, *Pto* DC3000 triggered higher MPK6 activation, with its maximum activity at an earlier time point compared with Col-0 (Fig. 3), whereas MAPK activities were abolished in the AP2C1-oe line. MAPK activities in the *mkp1* mutant were also more pronounced than in Col-0, but their kinetics was different than in *ap2c1* plants. Double mutant plants *ap2c1 mpk6* showed stronger activation of MPK3/4/11, and *ap2c1 mpk3* stronger activation of MPK6 compared with Col-0 (Fig. 3).

Taken together, our data demonstrate that during PAMP- and pathogen-induced responses AP2C1 plays a significant role in the negative regulation of MPK3, MPK4/11 and predominantly MPK6. AP2C1 deactivates MPK6 during early stages of bacterial invasion, whereas in the absence of AP2C1 a different phosphatase, such as MKP1, can effectively inactivate these kinases.

To investigate whether MPK3 or MPK6 plays a role in increased resistance observed in *ap2c1*, we analysed the double mutant lines *ap2c1 mpk3* and *ap2c1 mpk6* for plant resistance to *Pto* DC3000 and *Pto* DC3000 *ΔavrPto*/*ΔavrPtoB*. Absence of each of the MAPKs did affect the disease resistance as both double mutant lines demonstrated significantly enhanced resistance compared with the Col-0 plants (Fig. 1A, B). In response to *Pto* DC3000, *ap2c1 mpk6* plants showed significantly higher bacterial growth than the *ap2c1 mpk3* mutant, indicating a more substantial contribution of MPK6 to plant basal resistance in the *ap2c1* background than of MPK3 (Fig. 1A, B).

### Defense-related genes are deregulated in *ap2c1* and MAPK mutant plants

**Fig. 4.**
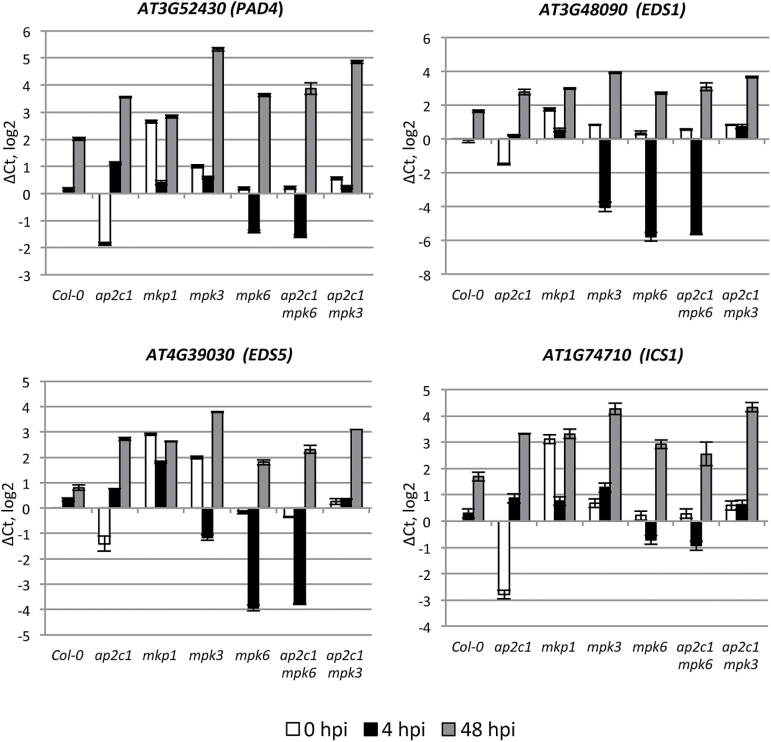
qRT-PCR analysis of SA-related gene expression. Adult 4-week-old plants were sprayed with *Pto* DC3000 or water as a mock control and harvested at 0 h (white bars), 4 h (black bars) and 48 h (2 dpi; gray bars) post-infection. The relative gene expression was normalized to the reference gene, *ACTIN2*. Results are from three biological and two technical replicates for each experiment. Error bars indicate SE. Values on the *Y*-axis are given on a log2 scale.

**Fig. 5.**
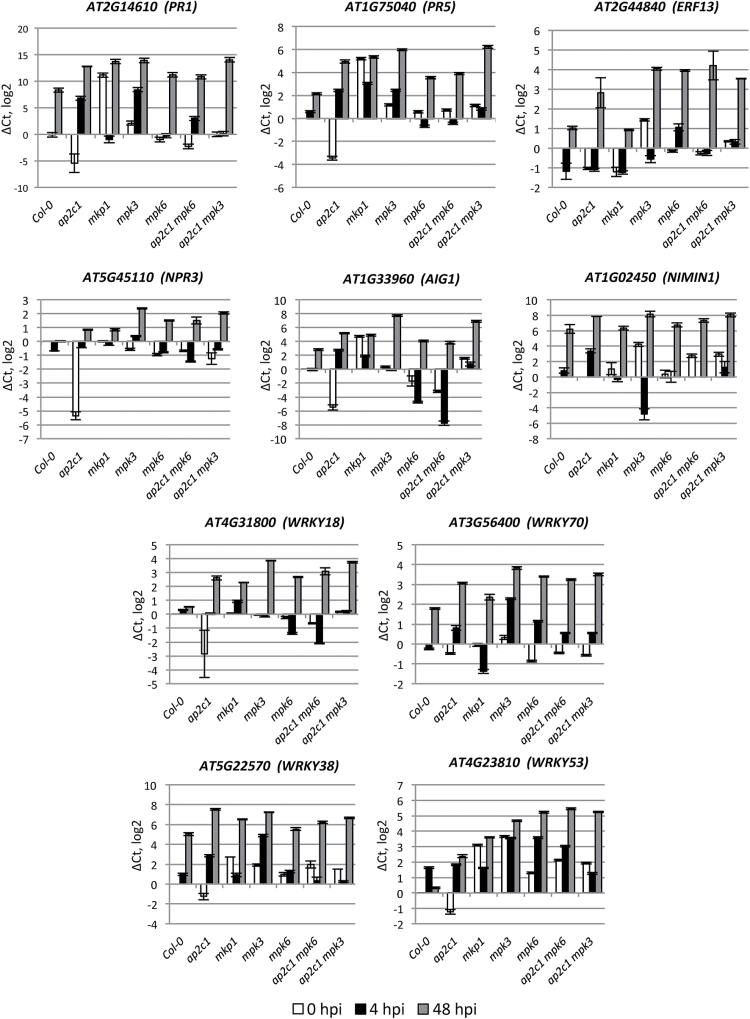
qRT-PCR analysis of defense-related gene expression. Adult 4-week-old plants were sprayed with *Pto* DC3000 or water as a mock control and harvested at 0 h (white bars), 4 h (black bars), and 48 h (2 dpi; gray bars) post-infection. The relative gene expression was normalized to the reference gene, *ACTIN2*. Results are mean of three biological and two technical replicates for each experiment. Error bars indicate SE. Values on the *Y*-axis are given on a log2 scale.

**Fig. 6.**
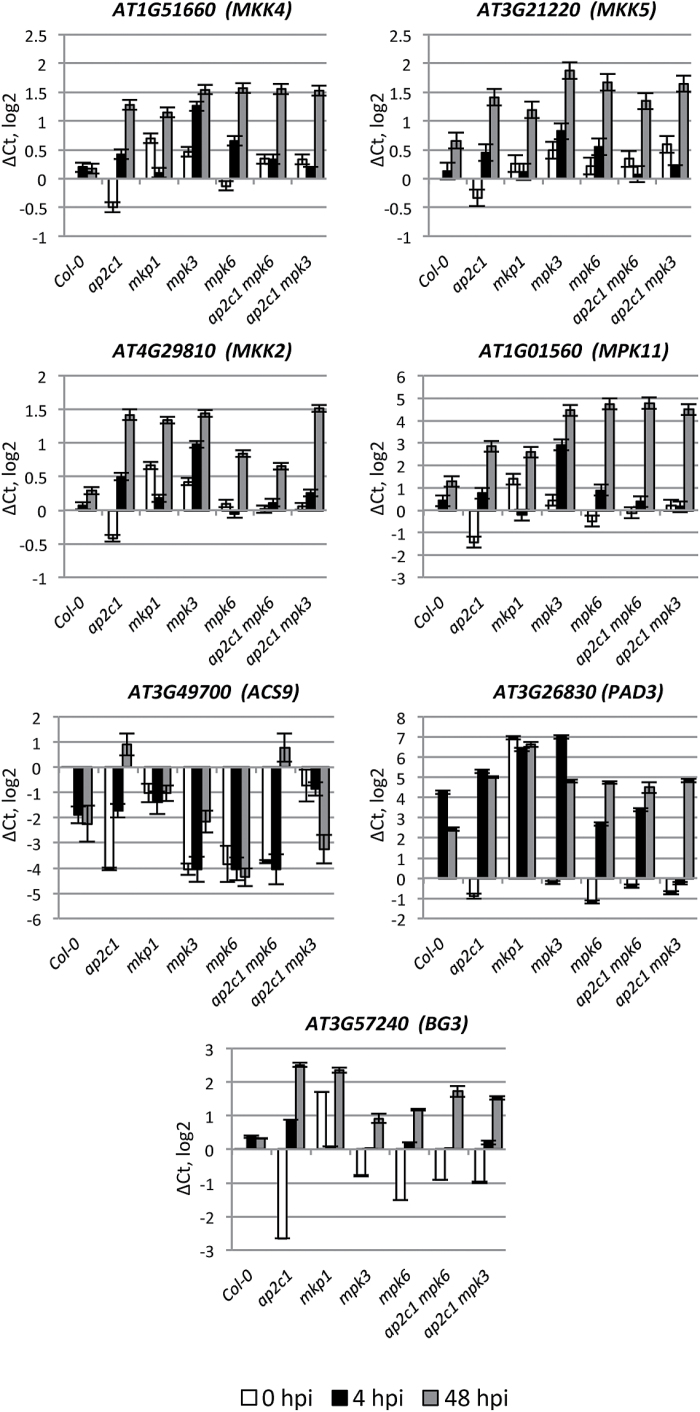
qRT-PCR analysis of pathogen-related gene expression (MAPK cascade components; ET-, camalexin- and callose-related genes). Adult 4-week-old plants were sprayed with *Pto* DC3000 or water as a mock control and harvested at 0 h (white bars), 4 h (black bars), and 48 h (gray bars) post-infection. The relative gene expression was normalized to the reference gene, *ACTIN2*. Results are mean of three biological and two technical replicates for each experiment. Error bars indicate SE. Values on the *Y*-axis are given on a log2 scale.

**Fig. 7.**
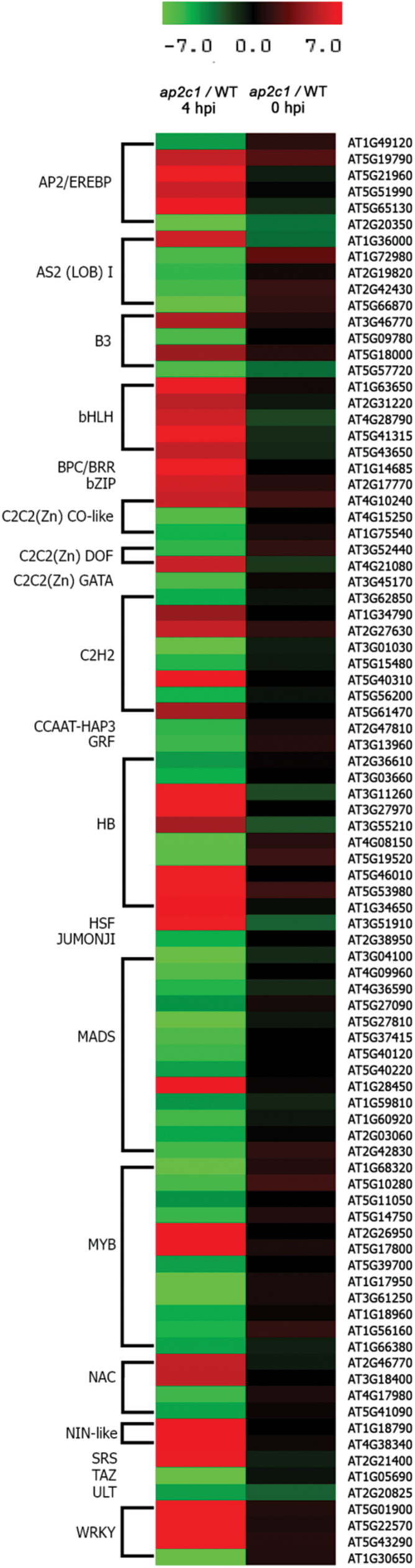
Heat map of 88 TF genes differentially regulated during *Pto* DC3000 immune response in *ap2c1* compared with WT plants. Expression levels were determined in leaves of treated plants by multi-parallel qRT-PCR analysis. Red and green indicate higher and lower expression values, respectively. Intensity of the colors is proportional to the absolute value of log2 of the difference in gene expression between *ap2c1* and WT. Black indicates no change in gene expression. Two biological replicates with two technical replicates in each were analysed.

To evaluate the impact of AP2C1 on pathogen responsive gene expression, we performed qRT-PCR of 137 defense-related genes (Brotman *et al.*, 2012). To cover ‘early’ and ‘late’ pathogen-induced gene expression, leaves were collected at 4 and 48 h post-infection (hpi) with *Pto* DC3000, respectively. Our data demonstrate significant deregulation (>1 on a log2 scale, which equals >2-fold) of genes involved in SA production (*EDS1*, *PAD4*, *EDS5*, and *ICS1*) and SA-induced genes, including *PR1*, *PR5*, *ERF13*, *PAD3*, *NPR3*, *AIG1*, and *NIMIN1* as well as transcription factors *WRKY18*, *WRKY70*, *WRKY53*, and *WRKY38*, in single and double *ap2c1 mpk3* and *ap2c1 mpk6* mutants compared with Col-0 (Figs 4–7 and Supplementary Fig. S3). Importantly, we observed a significant down-regulation of almost all genes listed above in untreated *ap2c1* plants. At 4 hpi we observed strong up-regulation of *PR1*, *PR5*, *PAD4*, *AIG1*, *WRKY70*, *WRKY38*, and *NIMIN1* genes specifically in *ap2c1* plants (Figs 4 and 5, and Supplementary Fig. S3).

In *ap2c1*, we also observed strong up-regulation of genes encoding components of the MAPK signaling cascade, such as *MPK11*, which is a flg22-activated gene (Bethke *et al.*, 2012), *MKK5* and *MKK4*, which are MAPKKs upstream of MPK3/MPK6 (Asai *et al.*, 2002), and *MKK2*, which is a positive regulator of plant immunity against *P. syringae*, acting upstream of MPK4/MPK6 (Brader *et al.*, 2007). *BETA-1*,*3-GLUCANASE 3* (*BG3*) (Dong *et al.*, 1991) and *ACS9*, which is involved in ET biosynthesis (Gomi *et al.*, 2005; Christians *et al.*, 2009; Tsuchisaka *et al.*, 2009), were also upregulated in comparison with Col-0 plants (Fig. 6 and Supplementary Fig. S3). However, *ACS9* expression was repressed in the *ap2c1 mpk3* line.

### Identification of AP2C1-regulated genes by genome-wide TF expression analysis

In order to identify possible links between enhanced activity of MAPKs and up-regulation of defense genes, we performed a genome-wide analysis of the expression of 1880 TF genes in Col-0 and *ap2c1*, using qRT-PCR. We found that 88 TF genes from 21 families, including basic helix–loop–helix (*bHLH*), *WRKY*, *NAC*, and *AP2/ERF* were differently regulated (>4 on a log2 scale) after 4 hpi with *Pto* DC3000 in *ap2c1 vs* Col-0 (Supplementary Table S1 and Fig. 7).

A promoter analysis of the 88 TF genes revealed enrichment of several motifs (Supplementary Table S2), such as CARGCW8GAT (8% increase over background), ARF binding site (8%), AtMYC2BSinRD22 (6%), MYCATERD1 (6%), L1-box motif (6%) and ATHB2 binding site (9%), suggesting these motifs as potential target sites for regulation by activated AP2C1-determined (MAPK) signaling. These results reveal the importance of AP2C1 as a regulator of many transcriptional responses, and suggest that many target genes are deregulated in the *ap2c1* mutant, thus altering plant immune responses after pathogen application.

### Induction of *AP2C1* and *AP2C2* expression by PAMPs and *Pto* DC3000

**Fig. 8.**
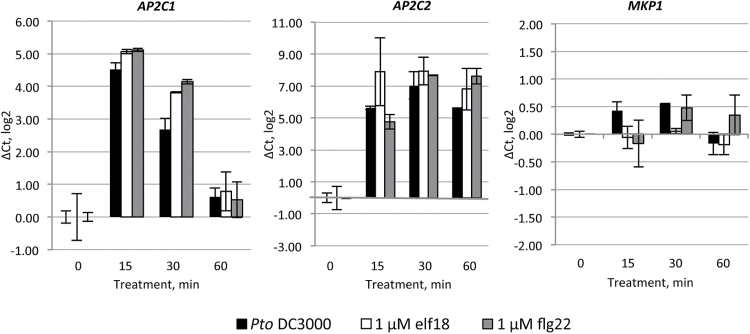
Gene expression of *AP2C1*, *AP2C2*, and *MKP1* after treatment with *Pto* DC3000, elf18 or flg22. Fourteen-day-old seedlings were treated with *Pto* DC3000, 1 µM elf18 or 1 µM flg22 and harvested at 0, 15, 30, and 60 min. The relative gene expression was normalized to the reference gene, *ACTIN2*. Results are mean of two biological and two technical replicates for each experiment. Error bars indicate SE. Values on the *Y*-axis are given on a log2 scale.

*AP2C1* and *AP2C2* have been detected as early PAMP-induced genes (Zipfel *et al.*, 2006) and the Genevestigator expression database (Hruz *et al.*, 2008) reports enhancement of their expression by PAMP treatment or during bacterial/fungal infection (see Supplementary Fig. S4). Monitoring the expression of *AP2C1*, *AP2C2* and other known or potential MAPK phosphatase genes in response to elf18, we observed differences in expression levels of these genes in seedlings upon a 180 min treatment with elf18 (Supplementary Fig. S5). *AP2C2* and *AP2C3* were significantly up-regulated upon treatment with elf18, whereas expression levels of other MAPK phosphatases, such as *AP2C1* and *AP2C4*, as well as of members from the PTP/DSP class were not significantly altered (Supplementary Fig. S5). To examine *AP2C1* and *AP2C2* expression at earlier time points, we performed qRT-PCR at 15, 30 and 60 min after application of *Pto* DC3000, elf18 or flg22 (Fig. 8). Strong *AP2C1* and *AP2C2* expression was detected already at 15 min after treatments. Interestingly, *AP2C1* transcript was no longer detectable at 60 min, whereas *AP2C2* demonstrated sustained expression, but *MKP1* expression was not altered (Fig. 8).

Collectively, the observation that both PAMPs and bacteria increase *AP2C1* and *AP2C2* expression in plants substantiates the idea of AP2C1 and AP2C2 as important players in plant response to PAMP and *Pto* DC3000.

### AP2C1 negatively controls accumulation of ET, but not camalexin or SA in response to PAMP or *Pto* DC3000

**Fig. 9.**
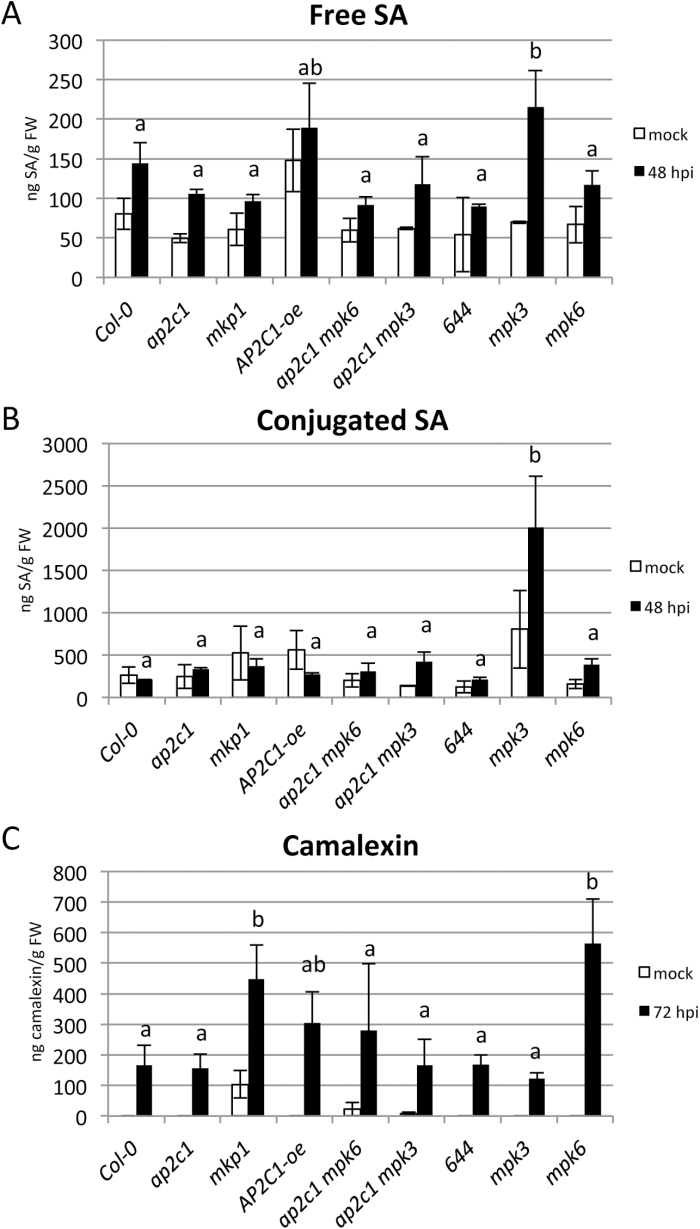
Analysis of SA and camalexin accumulation in plants treated with *Pto* DC3000. Levels of total free (A) and conjugated (B) SA, or total camalexin (C) as determined by HPLC in leaves of 4-week-old soil-grown plants mock spayed or treated with *Pto* DC3000 (OD_600_=0.02). (Results shown are mean±SE; *n*=4). One-way ANOVA/Holm–Sidak: *a*≠*b*, *P*<0.05.

Our analysis of defense gene expression indicated that SA and/or ET homeostasis might be modulated in the *ap2c1* mutant. Thus, we measured free and conjugated SA in plants at 24, 48 and 72 hpi with *Pto* DC3000. Our results show no significant differences in amounts of free or conjugated SA after pathogen application in *ap2c1* or *mkp1* mutant lines compared with Col-0, while these amounts increase in *mpk3* plants (Fig. 9A, B).

**Fig. 10.**
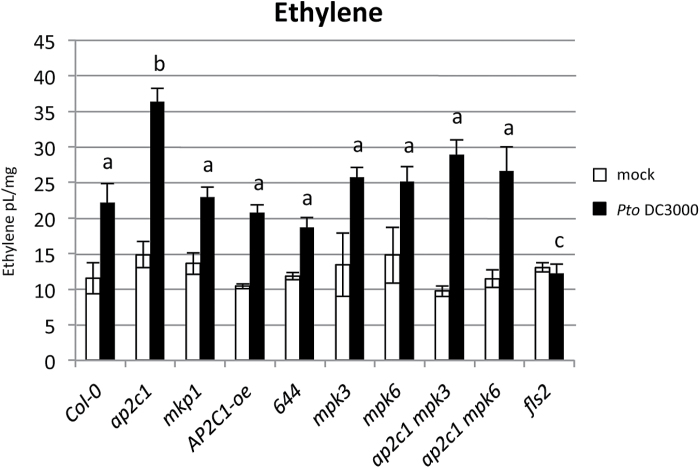
*Pto* DC3000-induced ethylene production in seedlings. Two-week-old seedlings of Col-0 and corresponding mutant lines were treated with *Pto* DC3000 (OD_600_=0.02) and total amount of ethylene produced by treated plants in 24 h was measured. (Results shown are mean±SE; *n*=6). One-way ANOVA/Holm–Sidak between treated line samples: *a*≠*b*, *P*<0.05; *a*≠*c*, *P*<0.02.

We measured ET levels after elf18, flg22 or *Pto* DC3000 treatments. Our results show no significant differences in ET amounts in all tested lines, except *efr* and *fls2* during elf18 or flg22 application, respectively (see Supplementary Fig. S6). However, in response to bacteria, *ap2c1* plants produce almost double the amount of ET as Col-0 and other lines tested (Fig. 10). The *ap2c1* complemented line 644 produced ET amounts similar to Col-0, suggesting that lack of AP2C1 leads to enhanced ET production.

Since an important part of plant pathogen defense responses is the production of the antimicrobial compound camalexin (3-thiazolylindole) (Meng and Zhang, 2013), we studied its accumulation in plant leaves after *Pto* DC3000 infection. Camalexin accumulation after bacterial inoculation was similar in Col-0 and *ap2c1*, while higher amounts of camalexin were produced in *mkp1* and *mpk6* in comparison to Col-0, *mpk3* and the corresponding double mutant lines (Fig. 9C).

### Callose deposition is enhanced in *ap2c1* seedlings in response to PAMP and *Pto* DC3000

**Fig. 11.**
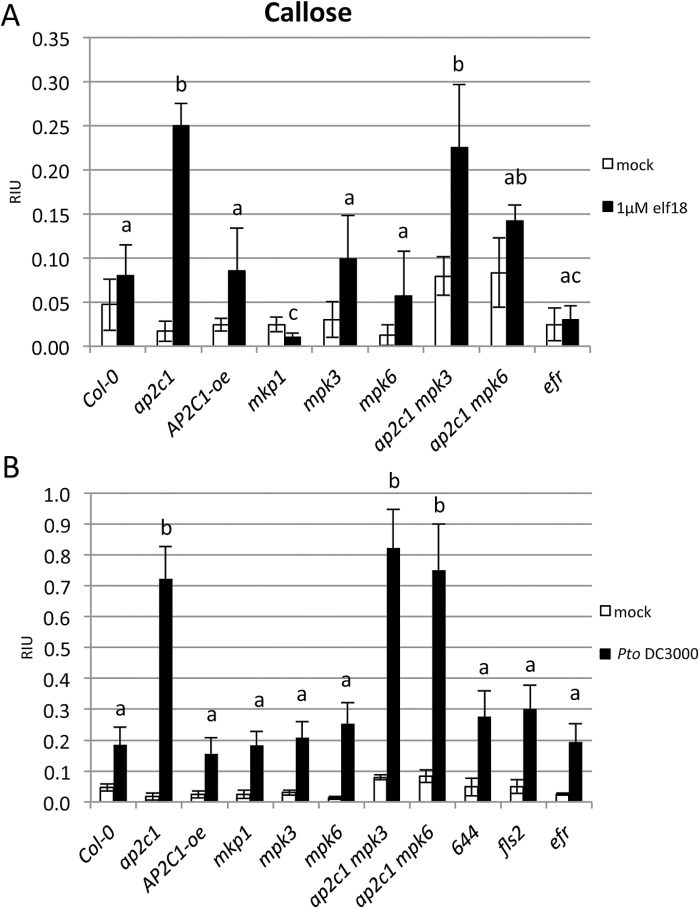
Callose deposition in cotyledons in response to elf18 or *Pto* DC3000. Ten-day-old seedlings were treated for 24 h with 1 µM elf18 (A) or *Pto* DC3000 (OD=0.02) (B). Photographs of aniline blue-stained cotyledons under UV epifluorescence were quantified with ImageJ. Data shown are mean values±SE (*n*>10) of relative callose intensities (RIU, relative intensity units) as measured at 24 h after pathogen or PAMP treatment. One-way ANOVA/Holm–Sidak between treated lines samples: *a*≠*b*, *P*<0.03; *a*≠*c*, *P*<0.05.

To measure callose accumulation in plants, we treated 2-week-old seedlings with 1 μM elf18 or *Pto* DC3000 for 24 h. Interestingly, plants lacking AP2C1 responded strongly to elf18 or *Pto* DC3000 by accumulating several times higher amounts of callose compared with Col-0 or AP2C1-oe plants (Fig. 11 and Supplementary Fig. S7). The enhancement of callose accumulation observed in *ap2c1* remained in *ap2c1 mpk3* and *ap2c1 mpk6* plants (Fig. 11). At the same time, the complementation of *ap2c1* mutation (line 644) showing a response similar to Col-0 indicated the reversion to the wild type phenotype (Fig. 11B). Corresponding to the previously published data (Anderson *et al.*, 2011, 2014) *mkp1* showed reduced callose deposition after PAMP treatment, and in response to *Pto* these plants responded similarly to Col-0.

## Discussion

Despite recent progress in understanding the role of signaling by MAPKs in plant immunity, less is known about their negative regulation by MAPK phosphatases. Focusing on the role of the MAPK phosphatase AP2C1, we have gained insights about the mechanism of negative regulation of MAPK signaling and its association with plant defense responses during PTI as well as plant resistance to pathogenic bacteria. We found that loss of AP2C1 leads to strongly enhanced resistance to *Pto* DC3000 that correlates with enhanced PAMP- and *Pto*-induced MAPK activities displaying particular kinetics, specific transcriptional reprogramming after *Pto* inoculation, and enhanced accumulation of ET and callose in infected plant tissues.

Additionally, our study pinpoint the specificity of MAPK regulation by the MAPK phosphatases AP2C1 and MKP1, which control the same substrate MAPKs, but differentially regulate the amplitude or timing of the kinase activities and related downstream events. These MAPK phosphatases evolved independently and utilize different dephosphorylation mechanisms (Brautigan, 2013). Importantly, the distinctive control of MAPKs by AP2C1 and MKP1 clearly correlates with the differences in plant pathogen responses. We suggest that, during challenge with pathogenic bacteria, individual negative regulators precisely and specifically control defined MAPKs to influence plant resistance. This is supported by our results demonstrating stronger resistance to *Pto* DC3000 in *ap2c1* compared with *mkp1* plants, which is likely due to a different mode of MAPK activation. We found that AP2C1 is a major determinant for plant sensitivity to *Pto*, acting in the earlier phase of kinase activation by flg22 or *Pto*, whereas the later phase is controlled by MKP1. Although enhanced resistance to *Pto* DC3000 was reported for *mkp1* (Col-0) (Bartels *et al.*, 2009; Anderson *et al.*, 2011, 2014), in our conditions these plants showed a similar disease resistance phenotype as Col-0. *Pto* DC3000 effectors AvrPto and AvrPtoB intercept signaling upstream of MAPKKK (He *et al.*, 2006) by interaction with FLS2, EFR, and BAK1 receptors (Göhre *et al.*, 2008; Shan *et al.*, 2008; Xiang *et al.*, 2008; Gimenez-Ibanez *et al.*, 2009*a*; Cheng *et al.*, 2011), and thus bacteria lacking these factors are less virulent. Interestingly, strong resistance of *ap2c1* plants to *Pto* DC3000 was reduced but still significant to isogenic hypovirulent strain *ΔavrPto/ΔavrPtoB*, whereas *mkp1* resistance was significantly enhanced, suggesting differences in control of plant resistance to bacterial infection for these MAPK phosphatases.

The closely related PP2Cs *AP2C1* and *AP2C2* are induced by PAMPs and pathogens, but their expression pattern differs: *AP2C1* expression is transient, whereas that of *AP2C2* is more sustained. AP2C1 plays a master role in controlling resistance to *Pto* DC3000; however, both AP2C1 and AP2C2 negatively regulate plant resistance to the less virulent *Pto* DC3000 strain COR^–^.

Strongly overlapping functions of MPK3 and MPK6, and lethality of their double mutants (Wang *et al.*, 2007) makes it difficult to decipher their individual roles in pathogen-induced cell signaling. MAPKK gain-of-function (GOF) approach helps to uncover the roles of MAPKs in defense responses (Meng and Zhang, 2013). The possibility of studying the mechanism of actions of specific MAPKs activated in their own domain is provided by T-DNA knock-out mutant plants of MAPK phosphatases (Schweighofer *et al.*, 2007; Bartels *et al.*, 2009; Anderson *et al.*, 2011, 2014). Here, studying the *ap2c1* mutant enabled us to reveal the roles of MPK3 and MPK6 in PTI. Genetic evidence emphasizes a strong positive role of MPK6 and also a contribution of MPK3 to PTI. Our results support the idea that activity of MAPKs, and mostly MPK6, which is regulated by AP2C1, is important for enhancement of plant resistance to *Pto* DC3000. Given the central role of MAPKs in signal transduction in response to PAMPs and *Pto* we showed that the precisely attenuated activity of MAPKs by a specific negative regulator in their native domain leads to a reduction of plant resistance. In addition, suppressed MAPK activities in response to PAMP and *Pto* and enhanced disease susceptibility due to *AP2C1* overexpression underline AP2C1 as a negative regulator of PTI and basal resistance. It correlates with the previous finding that *AP2C1* overexpression enhances plants sensitivity to the necrotrophic fungus *Botrytis cinerea* (Schweighofer *et al.*, 2007). In another report *AP2C1* over-expression revealed the major contribution of MPK6 in flg22-triggered resistance and contribution of MPK3 in basal resistance to *B. cinerea*, whereas no significant phenotypes were observed in *ap2c1* (Galletti *et al.*, 2011). In this study the robust plant resistance in the *ap2c1* and the enhanced sensitivity due to *AP2C1* overexpression demonstrate the importance of AP2C1 for negative regulation of plant immunity to the hemibiotrophic bacterium *Pto* DC3000.

Regulation of callose accumulation by cellular signaling is not well understood. It has been proposed that the activity of the callose synthase PMR4 might be regulated by post-translational mechanisms (Jacobs *et al.*, 2003; Ellinger *et al.*, 2013). Correlation of enhanced MAPK activation in *ap2c1* with enhanced deposition of *Pto*- and PAMP-induced callose suggests that the control of MAPK activation defines a possible mechanism in callose accumulation. Our findings are consistent with a previous report on enhanced callose accumulation by GOF MKK5, which activates MPK3/MPK6 (Zhang *et al.*, 2007*a*). It is tempting to speculate that PAMP- and *Pto*-triggered callose accumulation may result from the activation of PMR4 by AP2C1-defined MAPK signaling, since our results suggest a specific signal derived from MAPK activation towards callose accumulation. This feature is characteristic for AP2C1-controlled MAPK pathways, as *mkp1* shows strongly reduced callose accumulation in response to elf18, even though the *mkp1* mutant exhibits PAMP/*Pto*-induced MAPK activities. It can be speculated that higher activities of both MPK3 and MPK6 are required, possibly at specific locations in plant tissues or intracellular pools. In summary, our findings suggest that MAPK activities, which are enhanced due to the lack of AP2C1 in response to PAMP or *Pto*, control callose accumulation. Since callose is thought to act as a physical barrier to pathogen invasion (Ham *et al.*, 2007; Luna *et al.*, 2011), enhanced deposition of callose in *ap2c1* may explain the increased plant resistance to *Pto* DC3000 in this mutant.

Previously we reported reduced wound-induced ET production due to *AP2C1* overexpression, whereas *ap2c1* plants showed no significant difference to Col-0 (Schweighofer *et al.*, 2007). Here, in response to *Pto*, strongly upregulated ET amounts and enhanced kinase activities in *ap2c1* corroborate MPK6/MPK3-coordinated ET biosynthesis (Meng and Zhang, 2013). Intriguingly, elf18 and flg22 do not additionally enhance ET amounts in *ap2c1* plants, but *Pto* does, suggesting that recognition of bacteria by other plant receptors is integrated at the MPK3/MPK6–AP2C1 module. Notably, *mkp1* shows no additional enhancement of ET in response to *Pto* suggesting that an AP2C1-specific process of MAPK control is involved in regulation of ET amounts in response to bacterial infection. Importantly, study of *ap2c1* plants indicates that enhanced ET production and plant resistance to pathogenic bacteria are connected events. The observed repression of *Pto*-induced *ACS9* expression (Tsuchisaka *et al.*, 2009) in the *ap2c1 mpk3* line is evidence for a potential link for the proposed role of AP2C1 in MPK6-mediated regulation of ET biosynthesis (Liu and Zhang, 2004; Schweighofer *et al.*, 2007).

High-throughput methods for gene expression indicated a number of TFs playing key regulatory functions in pathogen responses (Chen *et al.*, 2002; Czechowski *et al.*, 2004; Caldana *et al.*, 2007); however, relatively few have been characterized as downstream targets of MAPK signaling (Andreasson and Ellis, 2010; Meng and Zhang, 2013). During *Pto* DC3000 infection, a contribution of AP2C1 was shown for 88 out of 1880 Arabidopsis TFs. This enables us to link transcriptional changes to the modulation of MAPK signaling and plant responses to pathogens. We observed a substantial and prevalent increase in expression of bHLH, basic region/leucine zipper motif (bZIP), AP2/ERF, NAC, and WRKY family members, whereas a predominant reduction for LOB(AS2), MADS, and MYB TFs indicates that loss of AP2C1 strongly affects TF transcript abundance during pathogen-induced signaling. Correlation of enhanced plant resistance with alterations in the expression of 88 TFs suggests their role in PTI and an impact on disease resistance. The identified TFs might be indirect downstream targets of MAPK signaling; a possible scenario is that the MAPKs control regulatory proteins, including perhaps TFs directly binding to the *cis*-regulatory elements enriched in the promoters of the TF genes affected in the *ap2c1* mutant (see Results), and that this control is affected by AP2C1. Significant transcriptional changes of defense-related genes indicate the involvement of a cell signaling cascade in their regulation. Strong downregulation of *PR1* and *PR5* in untreated *ap2c1* plants, and high upregulation at 4 hpi with *Pto* is remarkable, even though after infection a negative regulator of NPR1-mediated *PR* gene induction, namely *NIMIN1* (Weigel *et al.*, 2005), is upregulated. Since no significant changes of SA amounts were observed in pathogen-treated plants, it is likely that SA-independent mechanisms are activated in *ap2c1*, such as the one suggested to be induced by sustained MPK3/MPK6 activation (Tsuda *et al.*, 2013). Different roles of AP2C1 and MKP1 regarding the control of *PR1* expression are indicated by very high *PR1* levels in untreated *mkp1* confirming a previous report (Bartels *et al.*, 2009), although in our conditions the SA amounts were similar to Col-0. AP2C1 is required to maintain the basal expression levels of several defense-related genes. Downregulation of *AIG1*, *BG3*, *EDS1*, *EDS5*, *ICS1*, *PAD3*, *PAD4*, *PR1*, *PR5*, *WRKY38*, and *WRKY53* during ambient conditions in *ap2c1* and their upregulation in *mkp1* compared with Col-0 plants indicate different cellular roles of AP2C1 and MKP1. The upregulation of *WRKY38*, known to function as a negative regulator of plant basal defense (Kim *et al.*, 2008), in *ap2c1* compared with Col-0 or *mkp1* mutant plants, and the different regulation of *WRKY70* demonstrate that these MAPK phosphatases control PTI responses differentially. AP2C1 also plays a different role from MKP1 as part of a regulatory mechanism for several MAPK(K)s, since their expression is downregulated in untreated conditions in *ap2c1* plants. Changes in TF and defense responsive gene expression may contribute to or trigger enhanced *Pto* resistance of the *ap2c1* plants.

Analyses of the single MAPK mutants revealed differential control by MPK3 and MPK6 of the expression of several defense genes and the double mutants *ap2c1 mpk3* and *ap2c1 mpk6* indicated the expression of *PAD4*, *ICS1*, *PR5*, *NPR3*, *AIG1*, *WRKY18*, and *BG3* as MPK3 and MPK6 dependent, but epistatic to AP2C1.

In summary, our data reveal that MPK3 negatively regulates *PR5*, *PR1*, *WRKY38*, *WRKY53*, *PAD4*, *MPK11*, and *ICS1*, most probably via an AP2C1-controlled pathway. We also found that expression of *PR5*, *PR1*, and *NIMIN1* is redundantly controlled by MPK3/MPK6, while MPK6 is not regulated by AP2C1 to control their expression. *WRKY18* is controlled by AP2C1 but is independent of MPK3 and MPK6. After *Pto* infection the pattern of gene expression becomes even more complex, making it challenging to dissect dependencies on the action of single genes.

Specific regulation of camalexin by MKP1-, but not by AP2C1-controlled MAPK pathways is indicated by enhanced camalexin amounts in *mkp1* but not in *ap2c1*, even though *PAD3* (Schuhegger *et al.*, 2006) and *PAD4* (Zhou *et al.*, 1998) required for camalexin biosynthesis are upregulated in both lines after *Pto* infection. Our data support a previous report that MKP1 negatively regulates camalexin biosynthesis by controlling MPK3 and MPK6 (Bartels *et al.*, 2009). Intriguingly, high upregulation of *PAD3* expression and SA amount in response to *Pto* in *mpk3* plants, and enhanced camalexin amounts in *mpk6* plants in comparison with Col-0 underline defined roles of these MAPKs in PTI. PAD4 and its interacting partner EDS1 function together to promote SA-dependent and -independent defenses (Feys *et al.*, 2001), and their regulation in *ap2c1* is MPK3 and MPK6 dependent. The fact that mutations in either of these MAPKs did not, or only partially, suppress enhancement of several defense responses implies their redundancy and suggests specific functions in signaling pathogen attack. This redundancy is illustrated by strongly enhanced remaining MPK6 activity in plants after elimination of MPK3 as it was observed previously in wound-induced signaling (Menke *et al.*, 2004).

In plant immunity the integration of perception of multiple non-self signals on the same conservative MAPK (Tena *et al.*, 2011; Rasmussen *et al.*, 2012; Meng and Zhang, 2013) indicates that regulation of specificity has to be ensured. Although RACK1 was recently identified as the first plant scaffold protein in protease-mediated but not flg22-mediated defense responses (Cheng *et al.*, 2015), regulation of both defense and developmental pathways by a MKK4/MKK5–MPK3/MPK6 module is puzzling, especially as MPK6 and MPK3 share a large percentage (40%) of tested substrates (Popescu *et al.*, 2009). Our results suggest that protein phosphatases bring precision in pathogen-activated MAPK control and this regulation may to some extent provide the specificity towards the downstream responses. An interesting question for future research is whether these negative regulators of MAPK activities act on the same pool of MAPKs or target MAPKs at different subcellular locations. Our previous results showed cytoplasmic and nuclear AP2C1 interaction with MPK6 (Schweighofer *et al.*, 2007), whereas Bartels *et al.* (2009) demonstrated an interaction of MKP1 with MPK6 predominantly in the cytoplasm. These phosphatases may therefore act on different subcellular pools of MAPKs.

It is obvious that the regulation of signaling pathways by protein phosphatases is complex. MAPK activities have to be kept under control by different MAPK phosphatases that exhibit different tissue expression patterns and subcellular localizations thereby collectively regulating signaling. It was shown that a coordinated action of MKP1 and PTP1 phosphatases is crucial for repression of plant SA-dependent autoimmune-like responses (Bartels *et al.*, 2009). A negative control mechanism of MAPKs maintains a highly conserved property to balance the activity of the upstream components with the downstream responses, as the duration and magnitude of MAPK activation play a major role in determining the biological outcome of signaling in animal cells (Caunt and Keyse, 2013).

Taken together, studying *ap2c1* plants has proved useful to connect this phosphatase with MPK3/MPK6-dependent endpoints, such as the activation of TF and pathogen-related gene expression, and ET and callose accumulation. Consequently, the proper spatial and temporal regulation of MAPK pathways by protein phosphatases may significantly contribute to specificity of signaling outcome. The future challenge is to elucidate the precise biological mechanism conferred by AP2C1 regulation of MAPK signaling and related plant responses.

## Supplementary data

Supplementary data are available at *JXB* online.

Fig. S1. Susceptibility of plants to *P. syringae.*

Fig. S2. Analysis of MAPK activation in plants by flg22.

Fig. S3. Heat map of pathogen-related gene expression during the immune response to *Pto* DC3000 infection.

Fig. S4. Expression analysis of *AP2C1* and *AP2C2* in response to pathogens and PAMPs using Genevestigator.

Fig. S5. Induction of MAPK-phosphatases upon elf18 treatment.

Fig. S6. PAMP-induced ethylene production in seedlings.

Fig. S7. Callose deposition in cotyledons in response to the PAMP elf18 or to *Pto* DC3000.

Table S1. Expression of transcription factors (TFs) in plants in response to *Pto* DC3000 treatment.

Table S2. Promoter region analysis of 88 selected TFs, which are significantly altered in *ap2c1* plants treated with *Pto* DC3000 in comparison to WT.

## Author contributions

VS, JB, JS, KK, MS, SB, and FB performed experiments; VS, AS, MS, FM, SB, BMR, FB, CZ, and IM designed experiments; and VS, JB, AS, FM, SB, BMR, FB, CZ, and IM wrote the manuscript.

## Supplementary Material

supplementary_figures_S1_S7_Tables_S1_S2
