# Supplementary material for: Protein phosphatase AP2C1 negatively regulates basal resistance and defense responses to Pseudomonas syringae
Source: J Exp Bot. 2017 Jan 6;68(5):1169–83. doi: 10.1093/jxb/erw485 (PMC5444444; doi:10.1093/jxb/erw485)
Supplement: supplementary_figures_S1_S7_Tables_S1_S2 [file erw485_suppl_supplementary_figures_S1_S7_Tables_S1_S2.pdf]

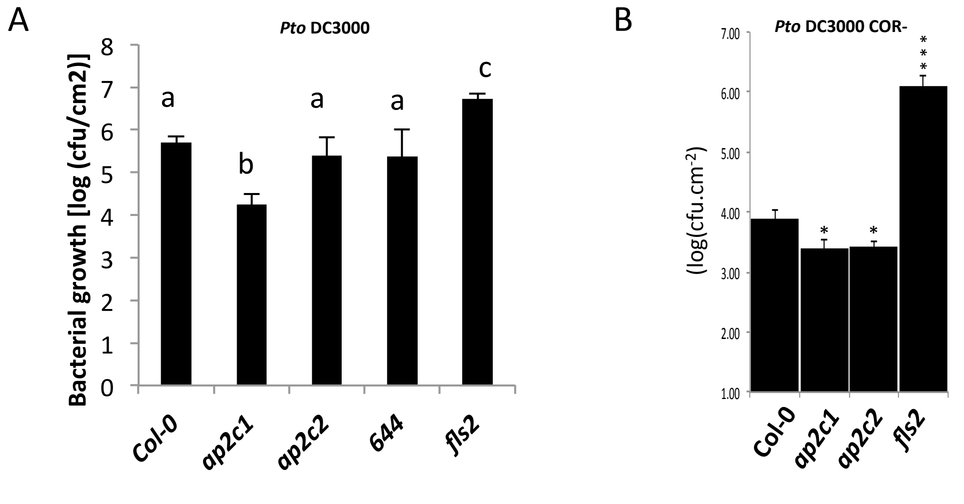

Figure S1: Susceptibility of plants to *P. syringae.*

A - Adult four-week-old plants were spray-infected with *Pseudomonas syringae* pv. *tomato* (*Pto*) DC3000 and bacterial count measured 4 d.p.i. 644: complemented line *ap2c1/AP2C1p::AP2C1-GFP* . One-way ANOVA/Holm-Sidak a≠b P<0.002, a≠c P<0.001.

B - Five-week-old plants were spray inoculated with *Pto* DC3000 *COR*$^{-}$ (OD_{600} = 0.02) and analyzed for bacterial growth at 3 d.p.i. Results are average ± SE (*n* = 6), performed with similar results two times.

Asterisks indicate: *p*<0.05 (*); *p*<0.005 (***) by *t*-test.

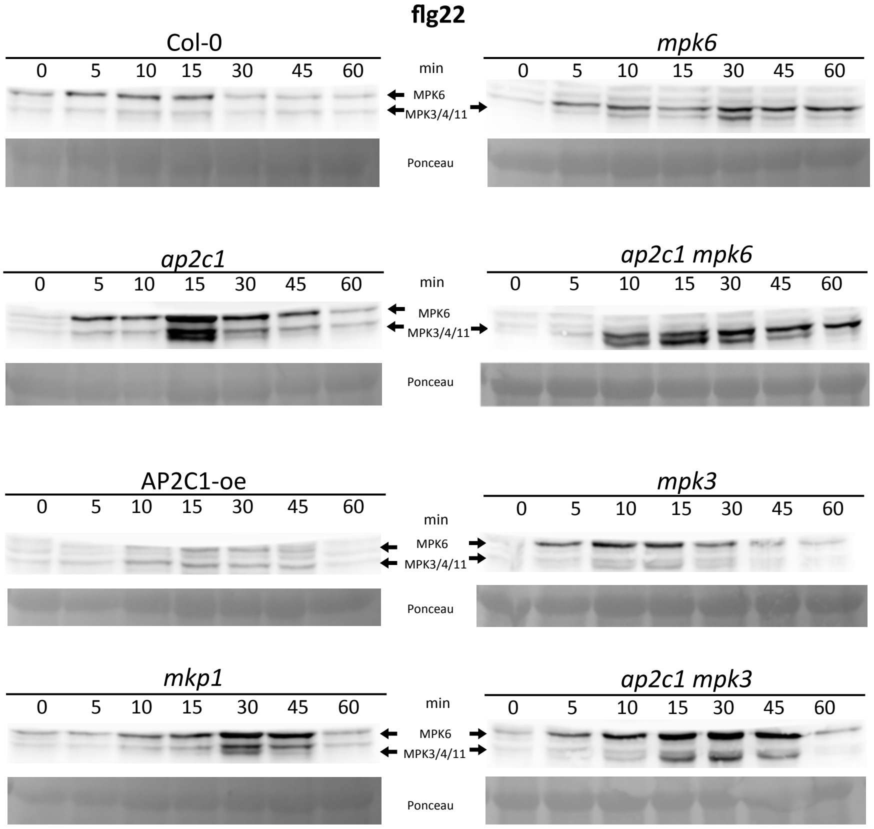

Figure S2. Analysis of MAPK activation in plants by flg22.

Western blotting with anti-p44/42 antibodies. PAMP activation of MAPKs in Arabidopsis seedlings of WT and modified lines after treatment with 1 µM flg22. The immunoreactive protein bands corresponding to respective MAPKs are indicated in the top panels, Ponceau staining was used to estimate equal loading (bottom panels).

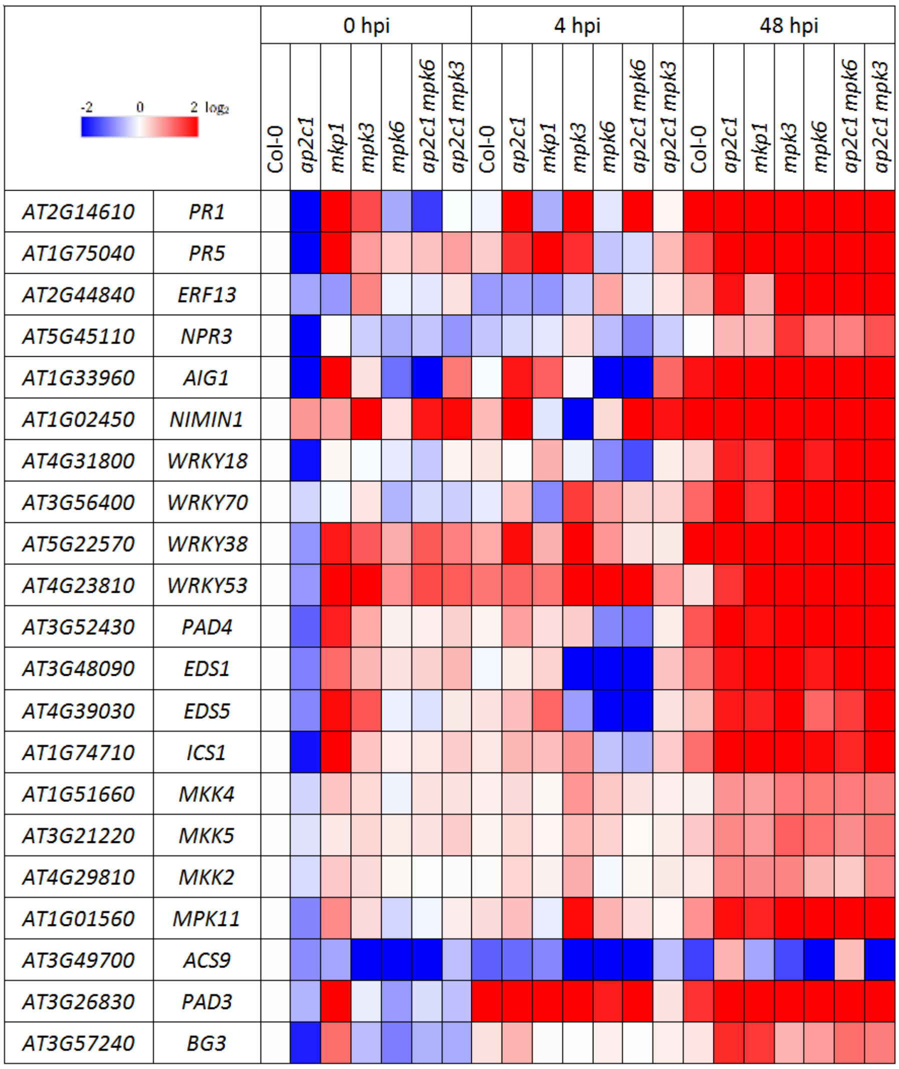

Figure S3. **Heat map of pathogen-related gene expression during the immune response to *Pto* DC3000 infection.** Expression levels were determined in leaves of treated plants by multi-parallel qRT-PCR analysis. Adult four-week-old plants were sprayed with *Pto* DC3000 or water as a mock control and harvested at 0, 4 and 48 hours post infection (hpi). The relative gene expression was normalized to the reference gene, *ACTIN2*. Blue and red indicate lower and higher expression values, respectively. Intensity of the colors is proportional to the absolute value of log2 of the gene expression difference compared to WT at 0 hpi. White indicates no change in gene expression compared to WT at 0 hpi. Results are mean of three biological and two technical replicates for each experiment.

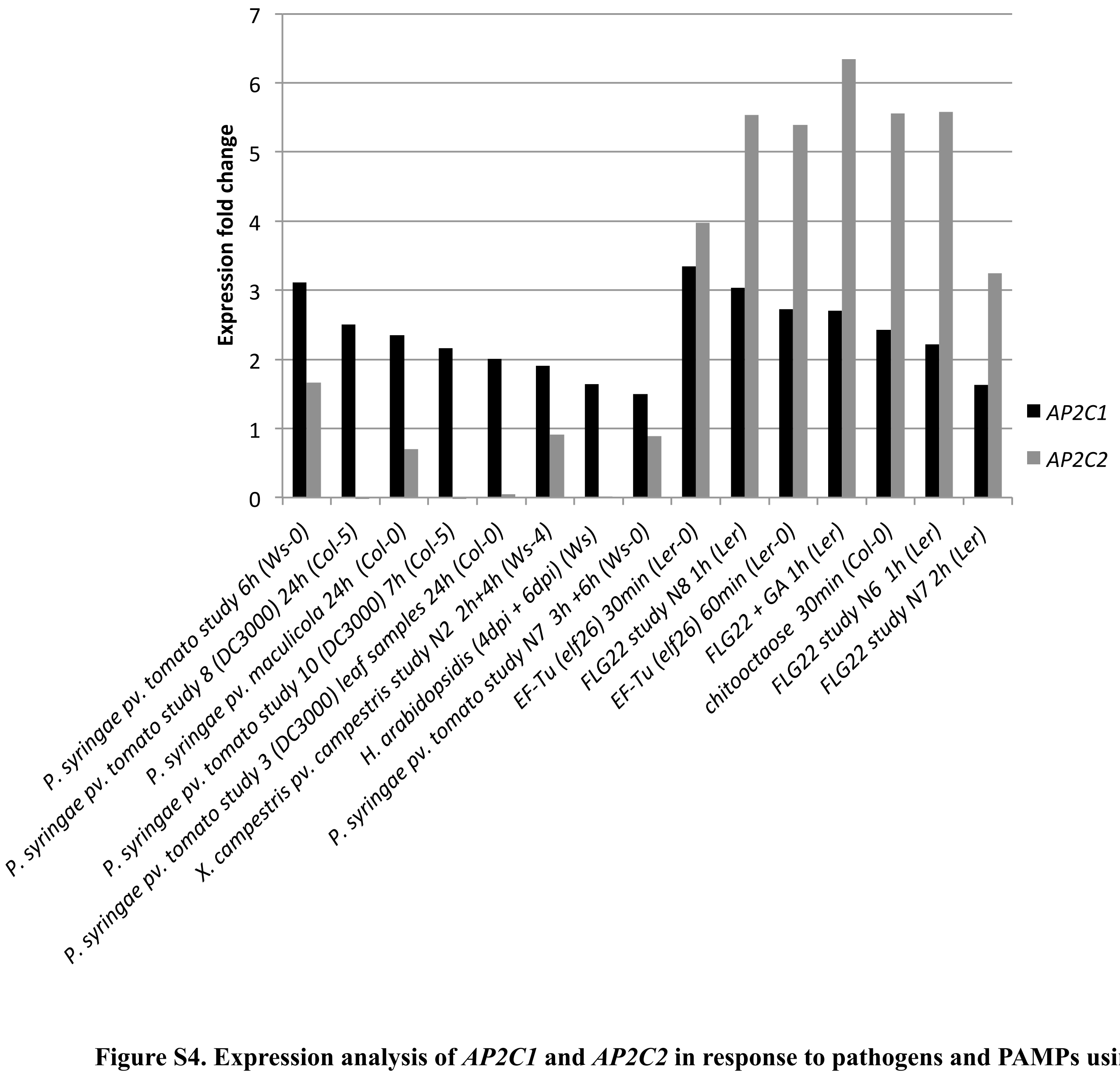

Figure S4. Expression analysis of *AP2C1* and *AP2C2* in response to pathogens and PAMPs using Genevestigator.

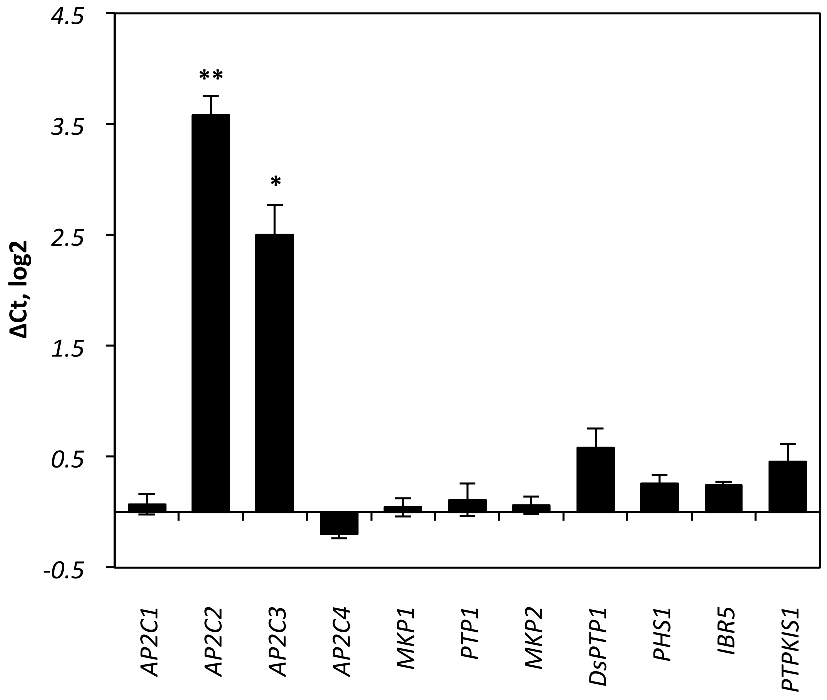

Figure S5. Induction of MAPK-phosphatases upon elf18 treatment.

qRT-PCR analysis of expression of MAPK-phosphatase genes in Col-0 14-day-old seedlings treated for 180 min with 100 nM elf18. Gene expression was normalized to expression of *ACTIN2* and plotted relative to non-treated expression levels in Col-0. Results are average of ± SE (n = 3), performed in two biological replicates. Asterisks indicate: p<0. 1 (*), p<0. 0.05 (**) by *t*-test.

Table S1. Expression of transcription factors (TFs) in plants in response to *Pto* DC3000 treatment. Selected members that are altered in *ap2c1* mutant compared to wild-type plants from analysis of 1880 TFs. Expression levels were determined by multi-parallel qRT-PCR and are shown as log2 changes of treated and untreated *ap2c1* mutant plant leaves compared to WT control. Two biological replicates with two technical replicates in each repetition were performed. The data are means of the two biological replicates, SE – standard error.

AGI	Gene family	ap2c1 / WT (4 hpi)	SE	ap2c1 / WT (0 hpi)	SE
AT1G49120	AP2/EREBP	-4,25	0,69	1,20	0,58
AT5G19790	AP2/EREBP	5,37	0,87	2,39	0,63
AT5G21960	AP2/EREBP	7,96	0,28	-0,61	0,81
AT5G51990	AP2/EREBP	5,58	0,49	-0,08	0,73
AT5G65130	AP2/EREBP	6,78	0,13	-1,19	0,15
AT2G20350	AP2/EREBP	-7,36	0,05	-3,13	0,78
AT1G36000	AS2 (LOB) I	5,67	0,37	-2,95	0,55
AT1G72980	AS2 (LOB) I	-5,86	0,31	2,78	0,48
AT2G19820	AS2 (LOB) I	-5,32	0,95	0,42	0,91
AT2G42430	AS2 (LOB) I	-5,76	0,94	1,70	0,19
AT5G66870	AS2 (LOB) I	-8,26	0,44	1,47	0,57
AT3G46770	B3	4,79	0,71	0,75	0,17
AT5G09780	B3	-5,97	0,45	0,00	0,38
AT5G18000	B3	4,29	0,92	0,99	0,87
AT5G57720	B3	-6,03	0,68	-2,94	0,78
AT1G63650	bHLH	6,54	0,60	0,44	0,23
AT2G31220	bHLH	5,13	0,48	-0,43	0,22
AT4G28790	bHLH	5,70	0,28	-1,91	0,13
AT5G41315	bHLH	7,66	0,01	-1,16	0,23
AT5G43650	bHLH	5,36	0,27	-0,94	0,21
AT1G14685	BPC/BRR	16,33	0,75	-0,03	0,66
AT2G17770	bZIP	5,85	0,14	1,02	0,80
AT4G10240	C2C2(Zn) CO-like	5,57	0,92	1,97	0,96
AT4G15250	C2C2(Zn) CO-like	-6,29	0,65	0,00	0,86
AT1G75540	C2C2(Zn) CO-like	-4,94	0,14	0,63	0,83
AT3G52440	C2C2(Zn) DOF	-5,27	0,49	1,48	0,08
AT4G21080	C2C2(Zn) DOF	5,54	0,66	-1,48	0,74
AT3G45170	C2C2(Zn) GATA	-5,92	0,97	0,28	0,02
AT3G62850	C2H2	-4,74	0,37	-0,31	0,33
AT1G34790	C2H2	4,15	0,54	0,00	0,09
AT2G27630	C2H2	5,44	0,02	1,36	0,73
AT3G01030	C2H2	-7,65	0,43	-0,63	0,84
AT5G15480	C2H2	-5,11	0,31	-0,45	0,79
AT5G40310	C2H2	6,65	0,07	-0,01	0,87
AT5G56200	C2H2	-4,93	0,73	-0,25	0,97
AT5G61470	C2H2	4,54	0,63	0,00	0,93
AT2G47810	CCAAT-HAP3	-5,29	0,94	0,63	0,62
AT3G13960	GRF	-5,51	0,27	0,98	0,11
AT2G36610	HB	-4,20	0,83	0,20	0,16
AT3G03660	HB	-4,82	0,01	0,00	0,49
AT3G11260	HB	7,42	0,04	-1,98	0,23
AT3G27970	HB	7,94	0,88	0,00	0,89
AT3G55210	HB	4,55	0,69	-2,20	0,41
AT4G08150	HB	-6,70	0,45	1,12	0,67
AT5G19520	HB	-6,55	0,71	1,82	0,01
AT5G46010	HB	7,02	0,05	0,00	0,15
AT5G53980	HB	6,74	0,66	-0,19	0,43
AT1G34650	HB	7,20	0,52	-2,62	0,93
AT3G51910	HSF	-4,81	1,00	-0,01	0,96
AT2G38950	JUMONJI	-7,20	0,73	-1,03	0,78
AT3G04100	MADS	-6,20	0,28	0,00	0,81
AT4G09960	MADS	-5,15	0,82	-1,05	0,44
AT4G36590	MADS	-4,03	0,72	0,53	0,74
AT5G27090	MADS	-7,33	0,87	-0,43	0,35
AT5G27810	MADS	-5,99	0,12	0,00	0,00
AT5G37415	MADS	-5,52	0,99	0,00	0,47
AT5G40120	MADS	-4,35	0,97	0,00	0,12
AT5G40220	MADS	6,68	0,41	0,23	0,82
AT1G28450	MADS	-4,08	0,25	-0,92	0,42
AT1G59810	MADS	-5,65	0,20	-0,38	0,61
AT1G60920	MADS	-4,54	0,59	-0,07	0,15
AT2G03060	MADS	-5,70	0,53	1,35	0,80
AT2G42830	MADS	-6,92	0,48	0,95	0,54
AT1G68320	MYB	-5,81	0,31	1,94	0,02
AT5G10280	MYB	-4,00	0,40	0,00	0,07
AT5G11050	MYB	-5,40	0,54	0,86	0,90
AT5G14750	MYB	6,90	0,84	0,00	0,67
AT2G26950	MYB	6,60	0,78	0,67	0,74
AT5G17800	MYB	-4,36	0,86	0,00	0,62
AT5G39700	MYB	-7,60	0,04	0,62	0,64
AT1G17950	MYB	-7,17	0,75	0,58	0,15
AT3G61250	MYB	-4,71	0,15	0,27	0,32
AT1G18960	MYB	-5,00	0,65	1,54	0,21
AT1G56160	MYB	-4,50	0,94	-0,69	0,66
AT1G66380	MYB	7,12	0,19	1,85	0,19
AT2G46770	NAC	5,35	0,20	-0,55	0,01
AT3G18400	NAC	5,32	0,71	0,01	0,24
AT4G17980	NAC	-5,55	0,32	0,72	0,75
AT5G41090	NAC	-4,52	0,14	0,31	0,82
AT1G18790	NIN-like	6,91	0,01	0,00	0,70
AT4G38340	NIN-like	6,94	0,82	0,36	0,17
AT2G21400	SRS	6,51	0,26	-0,74	0,17
AT1G05690	TAZ	-11,89	0,38	-0,28	0,69
AT2G20825	ULT	-4,36	0,25	-2,63	0,53
AT5G01900	WRKY	7,21	0,86	0,92	0,96
AT5G22570	WRKY	11,30	0,66	0,73	0,55
AT5G43290	WRKY	7,21	0,21	0,97	0,97
AT1G30650	WRKY	-7,07	0,63	0,83	0,69

Transcription Factor / Motif Name	prom's bound in subset		prom's bound in genome		p-value in genome
TATA-boxMotif	88%	77	82%	24789	0,001
MYB1AT	79%	69	85%	25733	0,145
MYB4bindingsitemotif	75%	66	75%	22642	0,012
CARGCW8GAT	67%	59	59%	18011	0,005
T-boxpromotermotif	54%	47	55%	16720	0,08
W-boxpromotermotif	54%	47	67%	20292	0,807
GAREAT	50%	44	55%	16578	0,327
ARFbindingsitemotif	48%	42	40%	12192	<10e-3
AtMYC2BSinRD22	41%	36	35%	10746	0,011
MYCATERD1	41%	36	35%	10746	0,011
Iboxpromotermotif	40%	35	40%	12259	0,084
BoxIIpromotermotif	34%	30	42%	12901	0,684
MYBbindingsitepromoter	24%	21	30%	9215	0,585
MYB2AT	22%	20	29%	8742	0,595
CCA1bindingsitemotif	20%	18	27%	8251	0,771
DREcoremotif	20%	18	23%	6989	0,336
L1-boxpromotermotif	20%	18	14%	4471	0,033
ATHB2bindingsitemotif	19%	17	10%	3181	0,005
ABRE-likebindingsitemotif	17%	15	20%	6258	0,641
CACGTGMOTIF	16%	14	15%	4546	0,277
MYB1LEPR	13%	12	17%	5127	0,619
AtMYB2BSinRD22	12%	11	12%	3757	0,248
Gap-boxMotif	12%	11	10%	3174	0,131
LEAFYATAG	11%	10	10%	3293	0,181
SV40corepromotermotif	11%	10	20%	6246	0,875
ACGTABREMOTIFA2OSEM	10%	9	14%	4398	0,809
GADOWNAT	10%	9	8%	2579	0,226

Table S2. Promoter region analysis of 88 selected TFs, which are significantly altered in *ap2c1* plants treated with *Pto* DC3000 in comparison to WT. Promoter motives identified according to Athena web-based research tool. TF binding frequency and enrichment for subselected promoters and TFs were calculated. Database crossreference for these transcription factors is provided as well as a statistical test for enrichment of binding activity within the set of selected promoters.

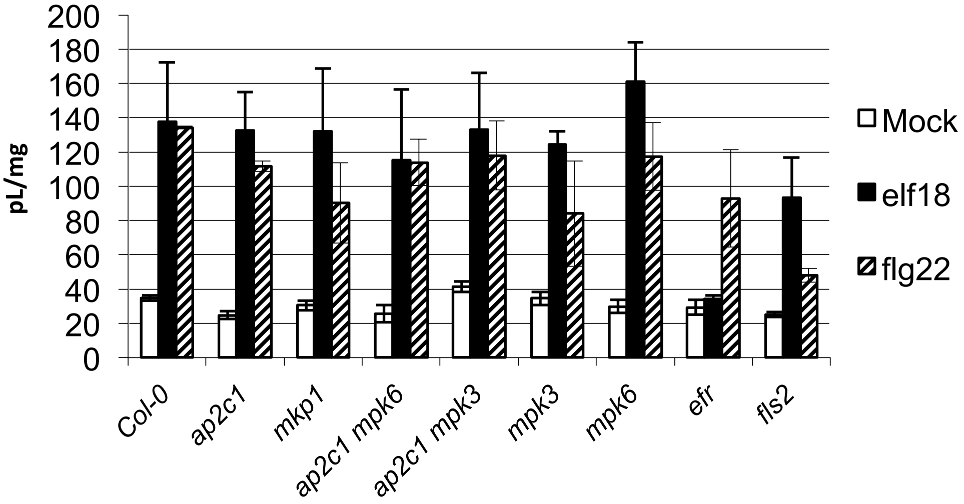

Figure S6. PAMP-induced ethylene production in seedlings.

Two-week-old seedlings of Col-0 and corresponding mutant lines were treated with 100 nM elf18 or flg22 and ethylene measurements were performed 24 hours after treatment. Results shown are average ± SE (n=6).

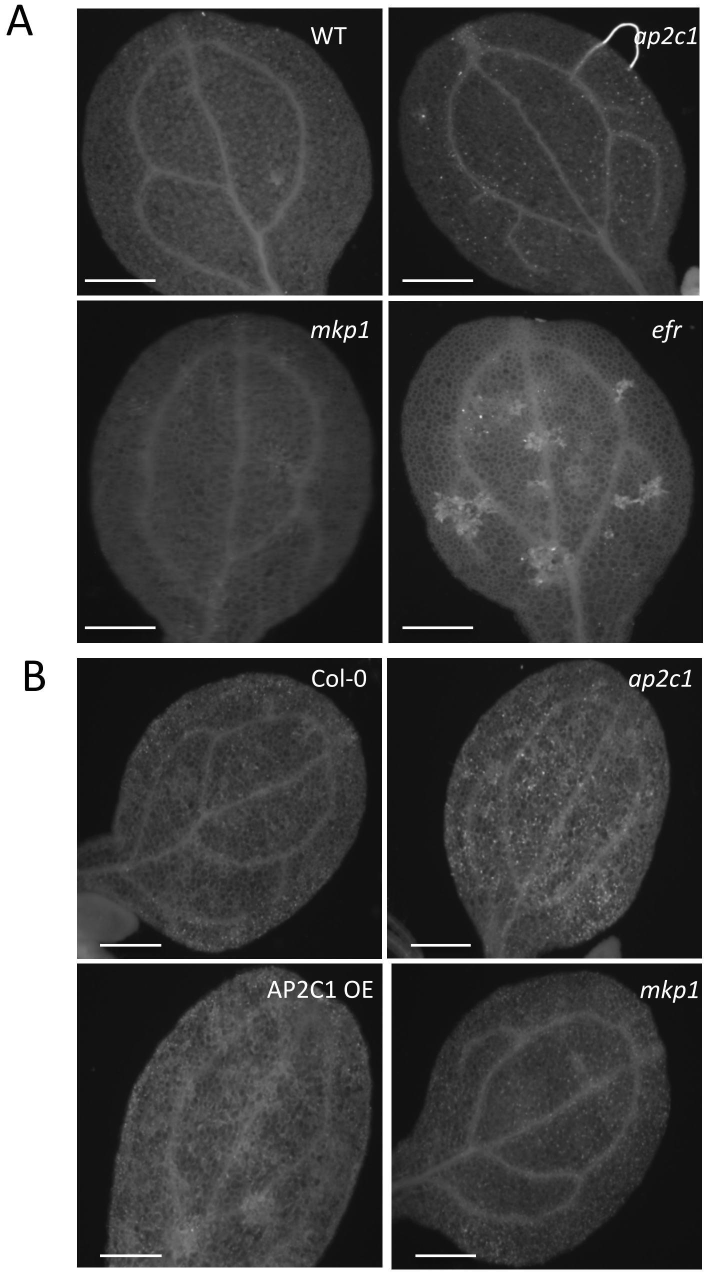

Figure S7. Callose deposition in cotyledons in response to the elf18 or to *Pto* DC3000.

Ten-day-old seedlings were treated for 24 h with 1 μM elf18 or with *Pto* DC3000 (OD_{600} = 0.02). Photographs of aniline blue–stained cotyledons under UV epifluorescence show morphological differences between the lines.

A – Callose deposition in response to 1 μM elf18; **B** - Callose deposition in response to *Pto* DC3000. Bar = 1 mm
